# Construct Validity of the EuroQoL–5 Dimension and the Health Utilities Index in Head and Neck Cancer

**DOI:** 10.1177/01945998211030173

**Published:** 2021-07-27

**Authors:** Christopher W. Noel, Sareh Keshavarzi, David Forner, Robert F. Stephens, Erin Watson, Eric Monteiro, Ali Hosni, Aaron Hansen, David P. Goldstein, John R. de Almeida

**Affiliations:** 1 Department of Otolaryngology–Head and Neck Surgery, Princess Margaret Cancer Centre–University Health Network, University of Toronto, Toronto, Canada; 2 Institute of Health Policy, Management and Evaluation, Dalla Lana School of Public Health, University of Toronto, Toronto, Canada; 3 Department of Biostatistics, University Health Network, Toronto, Canada; 4 Department of Otolaryngology–Head and Neck Surgery, Dalhousie University, Halifax, Canada; 5 Department of Dentistry, Princess Margaret Cancer Centre–University Health Network, University of Toronto, Toronto, Canada; 6 Department of Otolaryngology–Head and Neck Surgery, Sinai Health System, University of Toronto, Toronto, Canada; 7 Department of Radiation Oncology, Princess Margaret Cancer Centre–University Health Network, University of Toronto, Toronto, Canada; 8 Department of Medical Oncology, Princess Margaret Cancer Centre–University Health Network, University of Toronto, Toronto, Canada

**Keywords:** health state utility values, health-related quality of life, head and neck cancer, multiattribute utility instruments, preference-based measures, quality adjusted life years (QALYs)

## Abstract

**Objective:**

The objective of this study was to evaluate the construct validity of 2 health utility instruments—the EuroQoL–5 Dimension (EQ-5D) and the Health Utilities Index–Mark 3 (HUI-3)—and to compare them with disease-specific measures in patients with head and neck cancer.

**Study Design:**

Prospective cross-sectional analysis.

**Setting:**

Princess Margaret Cancer Centre.

**Methods:**

Patients were administered the EQ-5D, HUI-3, the European Organization for Research and Treatment of Cancer Quality of Life Questionnaire (EORTC QLQ-C30) and its head and neck cancer module (EORTC QLQ-H&N35), and the University of Washington Quality of Life Questionnaire (UWQoL). Several a priori expected relations were examined. The correlative and discriminative properties of the various instruments were examined.

**Results:**

A total of 209 patients completed the 4 questionnaires. A significant ceiling effect was observed among EQ-5D responses (23% reported a maximum score of 1). The EQ-5D (rho = 0.79) and HUI-3 (rho = 0.60) had a strong correlation with the social-emotional domain of the UWQoL. The EQ-5D had a moderate correlation with the physical domain of the UWQoL (rho = 0.42), whereas the HUI-3 had a weak correlation (rho = 0.29). The EQ-5D and HUI-3 were able to distinguish among levels of health severity measured on the EORTC QLQ-C30 though not the QLQ-H&N35. Comparatively, the UWQoL was able to distinguish levels of disease severity on the EORTC QLQ-C30 and QLQ-H&N35.

**Conclusion:**

The results of this study demonstrate that disease-specific domains from head and neck quality-of-life instruments are not strongly correlated with the EQ-5D and HUI-3. Consideration should be put toward development of a disease-specific preference-based measure for health economic evaluation.

**Level of evidence:**

4.

The treatment of head and neck cancer (HNC) involves surgery, radiotherapy, or chemotherapy, either independently or in combination. Treatments are toxic and carry significant health-related quality-of-life (HRQoL) implications.^1-5^ To assess HRQoL, several questionnaires specific to patients with HNC have been developed. Two of the most popular instruments are the European Organization for Research and Treatment of Cancer Quality of Life Questionnaire (EORTC QLQ-C30) and its Head and Neck Cancer Module (EORTC QLQ-H&N35)^6^ and the University of Washington Quality of Life Questionnaire (UWQoL).^7^ The EORTC and UWQoL have been shown to be valid and reliable for patients with HNC. They are capable of detecting even small changes in HRQoL.^6-11^ However, 2 key limitations exist. First, both questionnaires are specific to the HNC population and cannot be used across different diseases. Second, they do not provide health utilities (HUs). HUs are a universal measure of health outcomes and a key component of cost-effectiveness studies.^12,13^ HU scores are anchored between 1 (perfect health) and 0 (death). HUs are combined with length of time spent in that health state to generate quality-adjusted life years.

Several generic questionnaires have been developed that allow the HUs to be determined, including the EuroQoL–5 Dimension (EQ-5D) and the Health Utilities Index–Mark 3 (HUI-3).^14,15^ Despite the EQ-5D and HUI-3 being commonly used for many cancer sites, there has been limited uptake within head and neck oncology.^16-18^ Patients with HNC face unique toxicities, such as difficulties with swallowing, speech, and breathing. None of these symptoms are explicitly asked about in the EQ-5D or HUI-3. In other diseases, this lack of specificity has been shown to make the EQ-5D and HUI-3 nonresponsive to changes in HRQoL.^19-21^ This is problematic, particularly in the clinical trials space, where we are often comparing costly therapies that may lead to small but important differences in health status. Therefore, instruments that are able to generate HUs while being sensitive to change are desired.

Given this, we sought to better delineate the potential role of the EQ-5D and HUI-3 in HNC cost-effectiveness studies. Specifically, we aimed to evaluate the validity of the EQ-5D and HUI-3 by assessing whether these tools were able to distinguish across varying degrees of disease and treatment severity.

## Methods

### Patient Population and Setting

This validation study was conducted with prospectively collected data obtained from the Princess Margaret Cancer Centre, Toronto, Canada, between November 24, 2017, and March 23, 2018. HNC care in Ontario is provided through a universal single-payer health care system. Adult patients with mucosal squamous cell carcinomas of the upper aerodigestive tract were consecutively recruited from outpatient clinics. Non-English speakers and those who lacked decision capacity were excluded. Patients independently completed the EQ-5D, HUI-3, EORTC, and UWQoL in the same setting. Basic sociodemographic and clinical data were obtained.

### Instruments

The EQ-5D^14^ is a generic HU questionnaire that consists of items relating to mobility, self-care, usual activities, pain and/or discomfort, depression and/or anxiety, as well as a visual analog scale of the overall health status. We administered the 5-level version, which provides participants with a greater number of rating options as compared with the 3-level version. This has been shown to have reduced ceiling effects (fewer patients reporting the maximum score of 1).^14^ EQ-5D responses are transformed into utility scores (range, 0-1), which we performed using US population tariffs (preference weights derived from the US general population).

The HUI-3 is another generic HU questionnaire.^15^ It contains 8 questions/attributes pertaining to vision, hearing, speech, ambulation, dexterity, emotion, cognition, and pain and/or discomfort. Scores are assigned to each attribute and are combined with a formula to generate an HU score ranging from 0 to 1. The HUI-3 and EQ-5D allow for the generation of negative utilities, indicating a health state worse than death.

Version 4 of the UWQoL^7^ is an HNC-specific questionnaire based on 12 items, with each being scored on a scale from 0 (worst) to 100 (best). The physical and social-emotional domains are scored separately. The physical domain includes chewing, swallowing, speech, taste, saliva, and appearance. The social-emotional domain comprises anxiety, mood, pain, activity, recreation, and shoulder function. Domain scores are calculated through a mean score across items ranging from 0 to 100.

The EORTC QLQ-C30 is a cancer-specific quality-of-life questionnaire.^22^ It consists of 30 questions spanning key functional and symptom domains. The EORTC QLQ-H&N35 is an extension of the QLQ-C30 and is a module developed for use among patients with HNC.^6^ It consists of 35 items related to pain, swallowing, taste/smell, speech, social eating, social contacts, sexuality, teeth problems, trismus, dry mouth, sticky saliva, cough, and feeling ill. Unlike the UWQoL, an overall domain score does not exist.

### Determination of Validity

Validity is defined as the degree to which an instrument truly measures the constructs that it purports to measure.^23^ Two main types of validity testing exist: construct and criterion validity. Criterion validity assesses how the measure is compared against a gold standard, while construct validity tests expectations about how a measure should behave relative to hypotheses explicit to a conceptual framework.^24^ Owing to the absence of a gold standard in the measurement of HRQoL and health status, construct validity alone is typically measured. An early step in construct validity testing is to hypothesize how different measures should relate, also known as convergent construct validity.^25^ The more that an instrument behaves according to a priori hypothesized relations, the stronger the evidence for validity in that setting.^24^

We examined construct validity in several ways. We hypothesized that there would be a strong positive correlation between EQ-5D/HUI-3 and the physical and social-emotional domains of the UWQoL. As a person’s health status improves, so should one’s quality of life. Indeed, the UWQoL, EQ-5D, and HUI-3 all ask about mental health and physical functioning in various ways. We anticipated that there would be a strong positive correlation among these instruments. Unlike the UWQoL, the EORTC does not yield an overall score. Nonetheless, it does have several questions pertaining to attributes known to heavily influence HRQoL. We selected several of these questions a priori through a review of the literature and hypothesized that median EQ-5D and HUI-3 scores would be lower for patients reporting higher levels of symptom severity as compared with lower levels of symptom severity.^26^ We included UWQoL scores as a reference/comparator. Finally, we examined the EQ-5D, HUI-3, and UWQoL scores for patients who were <2 years from treatment completion, as compared with those who were ≥2 years from treatment completion. We hypothesized that HRQoL and HUs would be higher for those who were further from treatment completion.

### Statistical Analysis

Baseline characteristics were examined with descriptive statistics for the entire cohort. Categorical variables were reported as absolute number and proportion and continuous variables as either mean with standard deviation or median with interquartile range. Continuous variables were assessed for normality through the Shapiro-Wilk test and through visual inspection of the histogram and quantile-quantile plots.

The minimal clinically important difference for each HU instrument was calculated through a distribution-based method, which uses statistical criteria defined from the measurement results themselves, as opposed to external indicator “anchors.”^27^ We defined the threshold of discrimination and thus the minimal clinically important difference for each HU instrument as half of the standard deviation for the generated HU score.^28^

Spearman correlation coefficients were calculated to assess convergent construct validity between the UWQoL and the EQ-5D and HUI-3. Coefficients >0.60, 0.40 to 0.59, 0.21 to 0.39, and ≤0.20 were considered strong, moderate, weak, and no correlation, respectively.

The effect size, or standardized mean difference, between 2 groups on a measured outcome was also calculated. Responses from the EORTC were classified into meaningful comparator groups: *not at all*/*a little bit* or *quite a bit*/*very much*. The standardized mean difference describes the difference in means in units of standard deviation between 2 groups. It therefore allows us to directly compare the discriminative abilities of the EQ-5D, HUI-3, and UWQoL despite having different scales/variance.^29^ The absolute value of effect sizes were categorized as small (0.2-0.5), medium (0.5-0.8), or large (>0.8).^30^ Instruments that displayed larger effect sizes in a particular analysis were considered to have superior discriminative ability as compared with instruments displaying smaller effect sizes. Finally, we used the Mann-Whitney *U* test to compare EQ-5D, HUI-3, and UWQoL scores for those who were ≥2 or <2 years from treatment completion.

A 2-sided *P* value ≤.05 was considered significant. All analyses were performed with SAS University Edition (SAS Institute). The study received ethics approval from the University Health Network Research Ethics Board.

## Results

In total, 285 consecutive participants were approached and 209 agreed to participate (73%). Demographic characteristics are listed in **Table 1**. The majority of patients were men (72%) with a mean age of 63 years. The most common tumor sites were the oral cavity (35%) and oropharynx (25%). Relatively equal numbers underwent primary surgery and primary radiotherapy. Over 90% of patients had completed treatment and were being seen in surveillance.

**Table 1.** Demographic and Clinical Characteristics.

	Patients, No. (%)
Age, y	63 (18-97)^a^
Sex	
Female	59 (28)
Male	150 (72)
Race	
African American/Canadian	2 (1)
Asian or Pacific Islander	23 (11)
White	135 (66)
Hispanic	2 (1)
Indian or South Asian	13 (6)
Native American/Canadian	12 (6)
Other	18 (9)
Missing/refused	4
Education level	
Did not graduate high school	32 (15)
High school graduate	55 (27)
College graduate	78 (38)
Postgraduate degree	42 (20)
Missing or refused	2
Mean annual household income, CAD $	
<20,000	35 (19)
20,000-39,000	45 (24)
40,000-59,000	35 (19)
60,000-79,000	28 (15)
80,000-99,000	18 (10)
100,000-250,000	20 (11)
>250,000	8 (4)
Missing or refused	20
Marital status	
Never married	20 (10)
Married	135 (65)
Separated or divorced	29 (14)
Missing or refused	25 (12)
Site of primary tumor	
Oral cavity	73 (35)
Larynx	27 (13)
Oropharynx	52 (25)
Nasopharynx	4 (2)
Unknown primary	3 (1)
Hypopharynx	23 (11)
Parotid/major salivary	13 (6)
Nasal cavity	9 (4)
Other	5 (2)
AJCC stage, eighth edition	
0	3 (1)
I	50 (24)
II	54 (26)
III	26 (13)
IV	73 (35)
X	3
Treatment	
Surgery alone	42 (20)
Surgery + adjuvant radiotherapy	54 (26)
Surgery + adjuvant chemoradiotherapy	6 (3)
Salvage surgery	8 (4)
Radiotherapy alone	48 (23)
Chemoradiotherapy alone	49 (24)
Neoadjuvant chemotherapy	2 (1)
Treatment completed	
No	20 (10)
Yes	189 (90)
Time since completion of treatment, y	3.1 (4.1)^b^

Abbreviation: AJCC, American Joint Committee on Cancer.

a Median (range).

b Mean (SD).

**Table 2** reports the mean and median scores and the frequency of maximum scores (the ceiling effect). Ceiling effects were more common for the EQ-5D (23%) and the physical domain of the UWQoL (17%) than for the HUI-3 (7.7%) and the social-emotional domain of the UWQoL (9.6%). Based on a distribution-based method, the minimal clinically important difference of the instrument was 0.06 for the EQ-5D, 0.12 for the HUI-3, and 0.08 for the physical and social-emotional domains of the UWQoL. As the EORTC has no overall score, a minimal clinically important difference could not be calculated.

**Table 2.** Quality-of-Life Scores of the EQ-5D, HUI-3, and UWQoL.

Instrument	Mean	SD	Median	IQR	Minimum	Maximum	Ceiling, %
EQ-5D	0.84	0.12	0.83	0.78-0.88	0.352	1	23.9
HUI-3	0.72	0.25	0.79	0.58-0.79	−0.21	1	7.7
UWQoL							
Physical	80.4	15.7	83.3	70-8-91.7	28.3	100	17.2
Social-emotional	78.2	15.7	78.3	67.5-91.7	30.8	100	9.57

Abbreviations: EQ-5D, EuroQoL–5 Dimension; HUI-3, Health Utilities Index–Mark 3; IQR, interquartile range; UWQoL, University of Washington Quality of Life Questionnaire.

**Table 3** presents the correlations among the EQ-5D, HUI-3, and physical and social-emotional domains of the UWQoL. As anticipated, the EQ-5D (rho = 0.79) and HUI-3 (rho = 0.60) had a strong correlation with the social-emotional domain of the UWQoL. The EQ-5D had a moderate correlation with the physical domain of the UWQoL (rho = 0.42), whereas the HUI-3 had a weak correlation (rho = 0.29).

**Table 3.** Spearman Correlation Coefficients (Rho) for Global Scores Across the EQ-5D, HUI-3, and UWQoL.^a^

	EQ-5D	HUI-3	UWQoL: physical	UWQoL: social
EQ-5D	1			
HUI-3	0.68	1		
UWQoL: physical	0.42	0.29	1	
UWQoL: social-emotional	0.79	0.60	0.58	1

Abbreviations: EQ-5D, EuroQoL–5 Dimension; HUI-3, Health Utilities Index–Mark 3; UWQoL, University of Washington Quality of Life Questionnaire.

a *P* < .01 in all cases.

**Table 4** presents various relations between the EQ-5D, HUI-3, and UWQoL and selected items from the EORTC known to affect quality of life.^26^ As an example, a patient who answered *quite a bit* or *very much* on EORTC question 4 (“Do you need to stay in bed or a chair during the day?”) had an average EQ-5D score of 0.62 on a 0-1 scale. In contrast, a patient who answered *not at all* or *a little bit* on question 4 had an average EQ-5D score of 0.83. This corresponds to an effect size of 1.89 for the EQ-5D. Anything >0.8 is considered a large effect size.

**Table 4.** Relationship Between Selected Items From EORTC and the EQ-5D, HUI-3, and UWQoL.

	Score, mean (SD)			
	Not at all / a little bit	Quite a bit / very much	Effect size	*P* value
EORTC QLQ-C30				
**Q4: Do you need to stay in bed or a chair during the day?**				
EQ-5D	0.85 (0.11)	0.62 (0.14)	1.89	<.01
HUI-3	0.75 (0.22)	0.32 (0.30)	1.63	<.01
UWQoL				
Physical	80.8 (15.7)	74.8 (17.0)	0.37	.16
Social-emotional	79.6 (15.0)	58.9 (11.5)	1.54	<.01
**Q5: Do you need help with eating, dressing, washing yourself or using the toilet?**				
EQ-5D	0.84 (0.12)	0.51 (0.12)	2.71	<.01
HUI-3	0.73 (0.25)	0.34 (0.12)	2.00	<.01
UWQoL				
Physical	80.4 (15.9)	80.0 (8.2)	0.03	.97
Social-emotional	78.4 (15.4)	50.0 (22.4)	1.48	<.01
**Q6: Were you limited in doing either your work or other daily activities?**				
EQ-5D	0.86 (0.11)	0.69 (0.11)	1.46	<.01
HUI-3	0.75 (0.23)	0.50 (0.31)	0.94	<.01
UWQoL				
Physical	81.5 (15.2)	72.4 (18.2)	0.54	<.01
Social-emotional	80.4 (14.4)	61.4 (14.8)	1.30	<.01
**Q8: Were you short of breath?**				
EQ-5D	0.84 (0.12)	0.83 (0.13)	0.07	.85
HUI-3	0.72 (0.25)	0.56 (0.26)	0.67	.07
UWQoL				
Physical	80.5 (15.4)	76.7 (25.5)	0.18	.54
Social-emotional	78.4 (15.5)	72.9 (21.3)	0.30	.35
**Q9: Have you had pain?**				
EQ-5D	0.87 (0.10)	0.70 (0.12)	1.52	<.01
HUI-3	0.77 (0.22)	0.49 (0.26)	1.16	<.01
UWQoL				
Physical	81.6 (13.8)	61.9 (13.7)	0.38	.03
Social-emotional	61.9 (13.7)	75.8 (19.2)	1.43	<.01
**Q18: Were you tired?**				
EQ-5D	0.86 (0.11)	0.74 (0.13)	1.01	<.01
HUI-3	0.76 (0.22)	0.59 (0.31)	0.63	<.01
UWQoL				
Physical	82.2 (15.0)	73.6 (17.0)	0.53	<.01
Social-emotional	81.6 (13.9)	65.3 (15.4)	1.11	<.01
**Q22: Did you worry?**				
EQ-5D	0.86 (0.11)	0.73 (0.16)	0.98	<.01
HUI-3	0.76 (0.22)	0.46 (0.28)	1.23	<.01
UWQoL				
Physical	80.3 (16.3)	81.1 (12.9)	0.06	.80
Social-emotional	80.6 (14.5)	63.0 (14.6)	1.21	<.01
EORTC QLQ-H&N35				
**Q38: Have you choked when swallowing?**				
EQ-5D	0.84 (0.12)	0.81 (0.12)	0.27	.34
HUI-3	0.73 (0.24)	0.64 (0.33)	0.30	.21
UWQoL				
Physical	81.4 (15.3)	67.1 (17.6)	0.87	<.01
Social-emotional	78.8 (15.7)	70.6 (13.3)	0.56	.06
**Q39: Have you had problems with your teeth?**				
EQ-5D	0.85 (0.12)	0.78 (0.10)	0.67	<.01
HUI-3	0.74 (0.24)	0.63 (0.23)	0.50	<.01
UWQoL				
Physical	84.9 (12.1)	63.0 (16.4)	1.52	<.01
Social-emotional	81.1 (14.4)	67.0 (15.5)	0.94	<.01
**Q41: Have you had a dry mouth?**				
EQ-5D	0.86 (0.12)	0.80 (0.12)	0.49	<.01
HUI-3	0.74 (0.25)	0.69 (0.25)	0.20	.15
UWQoL				
Physical	86.5 (13.8)	72.8 (14.9)	0.96	<.01
Social-emotional	82.4 (14.7)	73.0 (15.3)	0.62	<.01
**Q42: Have you had sticky saliva?**				
EQ-5D	0.85 (0.12)	0.80 (0.13)	0.38	.01
HUI-3	0.74 (0.24)	0.68 (0.27)	0.21	.16
UWQoL				
Physical	84.6 (13.3)	69.7 (16.6)	0.99	<.01
Social-emotional	80.4 (14.8)	72.7 (16.4)	0.49	<.01
**Q53: Have you had trouble talking to other people?**				
EQ-5D	0.85 (0.12)	0.74 (0.12)	0.91	<.01
HUI-3	0.75 (0.24)	0.50 (0.25)	1.01	<.01
UWQoL				
Physical	83.0 (13.3)	58.4 (18.6)	1.52	<.01
Social-emotional	80.2 (14.8)	61.4 (12.7)	1.36	<.01
	No	Yes	Effect size	*P* value
**Q61: Have you taken painkillers?**				
EQ-5D	0.87 (0.11)	0.77 (0.13)	0.83	<.01
HUI-3	0.76 (0.23)	0.66 (0.26)	0.41	<.01
UWQoL				
Physical	82.7 (13.8)	76.3 (18.3)	0.39	.02
Social-emotional	83.2 (13.0)	69 (16.0)	0.96	.01
**Q62: Have you taken any nutritional supplements (excluding vitamins)?**				
EQ-5D	0.85 (0.13)	0.82 (0.12)	0.23	.10
HUI-3	0.73 (0.24)	0.70 (0.24)	0.11	.41
UWQoL				
Physical	83.1 (15.5)	76.1 (15.4)	0.45	<.01
Social-emotional	80.6 (15.3)	74.4 (16.0)	0.41	<.01
**Q63: Have you used a feeding tube?**				
EQ-5D	0.84 (0.12)	0.76 (0.12)	0.68	<.01
HUI-3	0.74 (0.25)	0.55 (0.23)	0.80	<.01
UWQoL				
Physical	82.4 (13.5)	56.1 (21.2)	1.48	<.01
Social-emotional	79.5 (15.5)	63.0 (8.3)	1.33	<.01
**Q64: Have you lost weight?**				
EQ-5D	0.85 (0.12)	0.79 (0.12)	0.46	.02
HUI-3	0.74 (0.25)	0.62 (0.24)	0.48	.01
UWQoL				
Physical	81.7 (15.7)	73.2 (14.4)	0.57	<.01
Social-emotional	79.8 (15.2)	69.6 (15.6)	0.66	<.01

Abbreviations: EORTC QLQ-C30, European Organization for Research and Treatment of Cancer Quality of Life Questionnaire; EORTC QLQ-H&N35, European Organization for Research and Treatment of Cancer Head and Neck Cancer Module; EQ-5D, EuroQoL–5 Dimension; HUI-3, Health Utilities Index–Mark 3; UWQoL, University of Washington Quality of Life Questionnaire.

Among the 7 selected questions from the generic EORTC QLQ-C30, the effects sizes between the *not at all*/*a little bit* and *quite a bit*/*very much* health states were large (>0.8) for all 3 instruments: UWQoL (7/7 questions), EQ-5D (6/7 questions), and HUI-3 (5/7 questions). Within the head and neck–specific module (QLQ-H&N35), the discriminative ability of the HU instruments was more limited. Whereas the effect sizes between dichotomized health states were large for the UWQoL (7/9 questions), large effect sizes were generated in only 2 of 9 questions for the EQ-5D (trouble talking, Q53; painkillers, Q61) and the HUI-3 (trouble talking, Q53; feeding tube use, Q63). For the EQ-5D and HUI-3, the generated effects sizes were small or nonsignificant for swallowing (Q38), dry mouth (Q41), sticky saliva (Q42), and nutritional supplementation (Q62). This suggests that these instruments have poor discriminative ability when attempting to differentiate varying levels of HNC disease severity. As expected, the median UWQoL, HUI-3, and EQ-5D scores were higher for those ≥2 years from treatment completion, though none of the results were significant to the predetermined *P* < .05 threshold.

## Discussion

Accurate HU elicitation is the cornerstone of health economics. National health agencies have stated the EQ-5D or HUI-3 should be preferentially used to measure HUs unless there is proof that these measures are not valid in the target population.^31^ This study demonstrates suboptimal construct validity for the EQ-5D and HUI-3 in the HNC population. While both measures correlate well with the social-emotional domain of the UWQoL and generic items of the EORTC QLQ-C30, they show moderate to weak correlation with the physical domain of the UWQoL and poor convergent validity with EORTC QLQ-H&N35. The EQ-5D is superior to the HUI-3 in that it has strong correlation with disease-specific quality-of-life measures and a tighter standard deviation leading to a smaller minimal clinically important difference. The EQ-5D is, however, limited by a more prominent ceiling effect.

Previous literature examining the validity of HU instruments in HNC is relatively sparse.^16^ Rogers et al demonstrated significant correlation between domains of the UWQoL and the EQ-5D in a cohort of 224 patients with HNC.^8^ Our group has shown that direct and indirect measures of HUs often produce disparate results and that the EQ-5D and HUI-3 (indirect measures) are better at distinguishing various measures of cancer severity relative to standard gamble and time trade-off (direct measures).^18^ We recently used the same data set from this study to generate a series of mapping algorithms to convert EORTC and UWQoL responses into EQ-5D and HUI-3 HU scores with ordinary least squares regression and 2-part models.^32,33^ The predictive performance of both algorithms was strong, though notably many of the HNC-specific items were not significant predictors of HUs on multivariable analysis.

Important differences exist between generic HU measures (EQ-5D and HUI-3) and disease-specific tools (EORTC and UWQoL). Because the EORTC and the UWQoL are focused on patients with HNC, more relevant and cancer-specific domains are included. From a clinical perspective, the EORTC and UWQoL offer superior face and content validity when compared with the EQ-5D and HUI-3.^34^ The EORTC and UWQoL appear to measure appropriate dimensions of HRQoL for patients with HNC (face validity) and have been well designed to include all important facets of HRQoL for this population (content validity). Disease-specific tools are, at times, required to identify relative differences in health status for economic evaluations in cases where generic HU measures are unable to do so. In this study, the EQ-5D and HUI-3 had suboptimal correlation with HNC disease-specific items (UWQoL physical and QLQ-H&N35), implying that these tools may not be able to detect all relevant differences in health status for this population. This is a concern, particularly in the context of clinical trials, where we are often attempting to distinguish between 2 modalities that may generate relatively small changes in health status. As an example, the NRG-HN006 compares sentinel lymph node biopsy with elective neck dissection in early oral cavity cancer and is collecting EQ-5D data as part of a planned cost-effectiveness study.^35^ It may be that the EQ-5D is unable to distinguish between these 2 health states, which would limit the investigators’ ability to generate accurate quality-adjusted life years.

The results of this study must be interpreted in the context of several key limitations. HU tariffs are region specific, and the results of this study might not generalize to other parts of the world. Specifically, the Canadian population and its ethnodemographic profile might not mirror other jurisdictions. Additionally, while we assessed convergent validity, we did not assess the responsiveness of the instruments, as this requires longitudinal assessment. Another limitation of this study is a possible selection bias because the questionnaires were distributed largely among survivors of HNC, potentially affecting the generalizability of our findings. Since the validity of the EQ-5D is in part limited by its prominent ceiling effect, its discriminative ability may improve in a broader HNC population. Additionally, there is an element of nonrespondent bias to this study for which we cannot readily adjust in our analysis. Finally, the EORTC QLQ-H&N35 was recently updated to the QLQ-H&N43. Several items were modified in this process, and results may not be directly transferrable.^36^

## Conclusion

Generic instruments such as the EQ-5D and HUI-3 are preferred by various national health agencies, although they run the risk of being unable to detect changes in the health status of certain patient populations. The EQ-5D and HUI-3 demonstrate suboptimal construct validity for patients with HNC. Head and neck oncology may benefit from a disease-specific preference-based measure for HU elicitation.
